# Emerging Roles of Long Noncoding RNAs in Immuno-Oncology

**DOI:** 10.3389/fcell.2021.722904

**Published:** 2021-11-25

**Authors:** Xin Wang, Xu Wang, Midie Xu, Weiqi Sheng

**Affiliations:** ^1^ Department of Pathology, Fudan University Shanghai Cancer Center, Shanghai, China; ^2^ Department of Medical Oncology, Shanghai Medical College, Fudan University, Shanghai, China; ^3^ Institute of Pathology, Fudan University, Shanghai, China

**Keywords:** lncRNA, cancer, tumor environment, immune surveillance, immuno-oncology

## Abstract

Long noncoding RNAs (lncRNAs), defined as ncRNAs no longer than 200 nucleotides, play an important role in cancer development. Accumulating research on lncRNAs offers a compelling new aspect of genome modulation, in which they are involved in chromatin remodeling, transcriptional and post-transcriptional regulation, and cross-talk with other nucleic acids. Increasing evidence suggests that lncRNAs reshape the tumor microenvironment (TME), which accounts for tumor development and progression. At the same time, the insightful findings on lncRNAs in immune recognition and evasion in tumor-infiltrating immune cells raise concerns with regard to immuno-oncology. In this review, we describe the essential characteristics of lncRNAs, elucidate functions of immune components engaged in tumor surveillance, and present some instructive examples in this new area.

## Introduction

In recent years, in both basic and clinical medical research, a popular and widely appreciated topic is elucidating the role of noncoding RNAs in human physiology and diseases. Decades ago, the major part of the genome, which does not encode proteins, was recognized as the “junk” DNA (Pennisi, 2012; McNally, 2017). Owing to the development of high-throughput sequencing technology, the GENCODE project has helped researchers to understand that ncRNAs cover over 90% of the human genome, and has annotated various kinds of ncRNAs, which promoted a widespread interest in long noncoding RNAs (Harrow et al., 2012; Frankish et al., 2019). The extensive studies of lncRNA have revolutionized our understanding of genetic and cellular molecule regulation in physiological and pathological phenomena.

An increasing number of studies have emphasized the view that lncRNAs are frequently involved in the process of cancer development. Controversy remains regarding the regulatory actions and effects of some lncRNAs in tumorigenesis. While in clinic translation, research on lncRNAs as a genetic screening marker or therapeutic target is ongoing (Do and Kim, 2018; Slack and Chinnaiyan, 2019). Meanwhile, immuno-oncology is becoming one of the most active fields in academic discovery. The abundant signal networks of immune cells, the multi-layered mechanisms of innate and adaptive immunity, and the numerous compositions of the tumor microenvironment (TME) add complexity to the explication of cancer immunity (Mayes et al., 2018; Weiden et al., 2018; Jones et al., 2019). We propose that, for immuno-oncology, besides the recognized protein-coding oncogenes and suppressors, studies on lncRNAs provide an important way to broaden our horizons in cancer immune biology.

In this review, we intend to summarize the definition and classification of lncRNAs considering their functions, to reveal the connections of lncRNAs with cancer immunity, and to highlight the relative translational research.

## Characteristics and Functions of lncRNA

**FIGURE 1:**
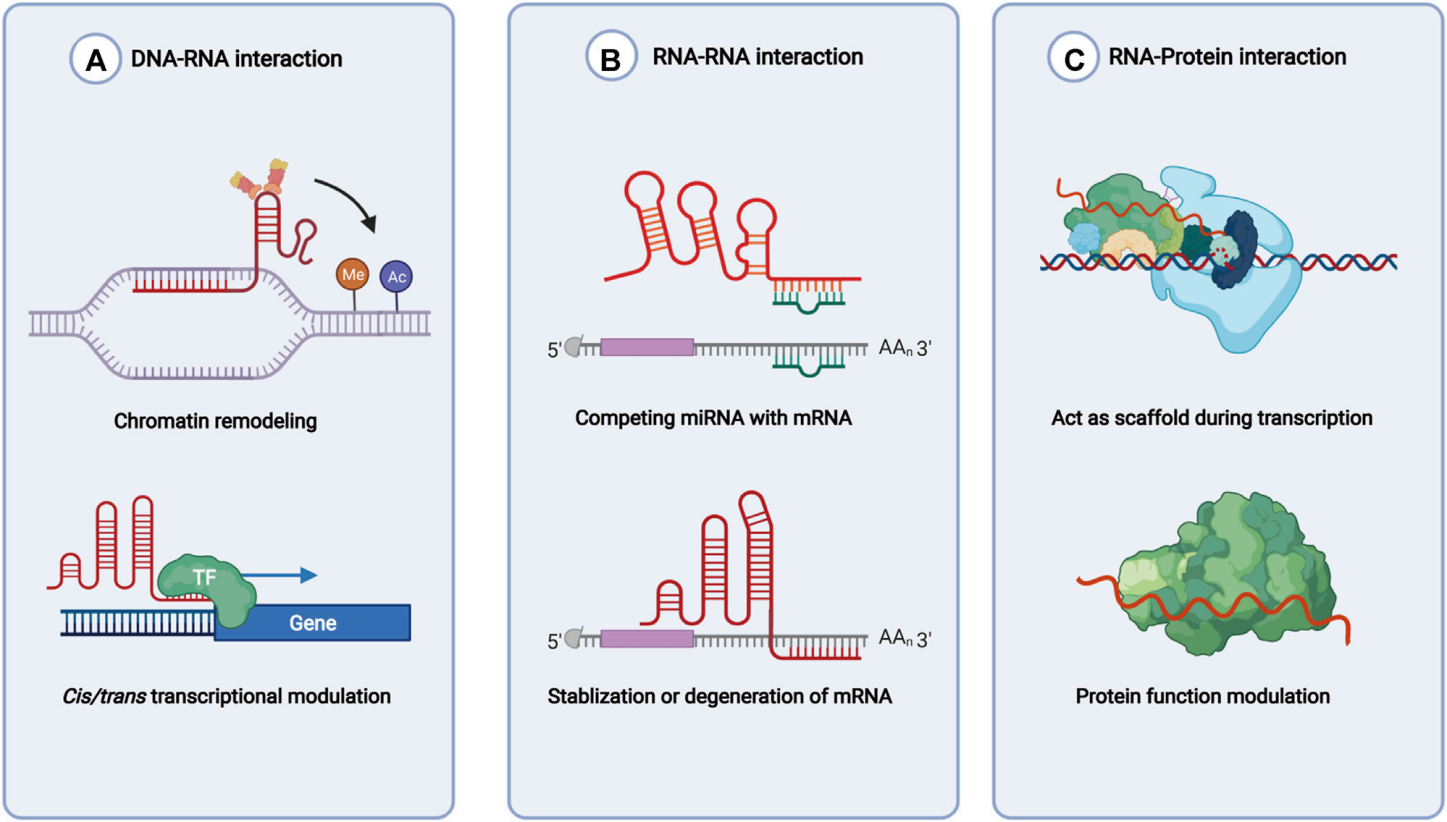
Functions of lncRNAs based on molecules interacted with. **(A)** lncRNAs recruit enzymes and transcription factors to involve in chromatin remodeling and transcription initiation. **(B)** lncRNAs regulate mRNA stability directly or indirectly. **(C)** lncRNAs bind with RNA binding protein and help with functions of the latter.

**FIGURE 2:**
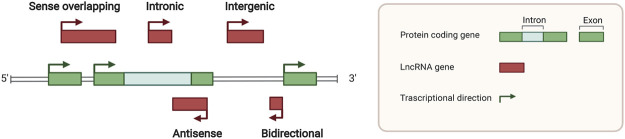
Locations of long noncoding RNAs in the genome. Green squares indicate protein coding genes in the genome, while red squares indicate noncoding RNA transcripts. Sense overlapping, antisense, intronic, intergenic, and bidirectional lncRNA loci was classified by different relative locations with protein coding genes.

lncRNAs are defined as the noncoding RNAs with a length of at least 200 nucleotides (nt). Compared with conventional protein-coding RNAs, lncRNAs are generally characterized as having lower abundance, more frequent nuclear location, and less evolutionary conservation (Ulitsky and Bartel, 2013; Ulitsky, 2016). For molecular structure, the mature polynucleotide strand is often 5’ capped, tailed with poly adenosine (Poly A) and/or spliced (Gruber and Zavolan, 2019). The functions of lncRNAs rely on their interactions with biomolecules, such as DNA (enhancer, promoter), proteins (transcription factor, enzyme) and other types of RNA (mRNA, miRNA) (Figure 1). Given their interactions with DNA, RNA, and proteins, several key technologies and tools are commonly used to identify and annotate their roles, such as ChiRP (Chu et al., 2012), RIP (Gagliardi and Matarazzo, 2016), RNA-FISH (Mahadevaiah et al., 2009), and PAR-CLIP (Spitzer et al., 2014). Notably, new evidence has convinced scientists that most transcripts of the annotated lncRNAs do not have direct biological functions (Uszczynska-Ratajczak et al., 2018). The functional activity of some transcripts is not related to their abundance in cells and the specific-sequence, which might be consistent with the above features. Given the discovery of bi-directional transcription initiation in promoters and enhancers, lncRNAs represent the major group of RNAs that are initiated at the DNA elements, which lacks encoding functions. According to the relative location to the coding-gene and the direction of transcription, lncRNAs are broadly classified as antisense, intronic, intergenic, sense overlapping, and bidirectional lncRNAs (Figure 2) (Harrow et al., 2012).

## Components and Events of Cancer Immune Recognition and Escape

The recent compelling achievements in cancer immune therapy are attributable to the persistent exploration of immunobiology during all stages of cancer development. It has been gradually appreciated that many, if not most, neoantigens from distinct cancer cells can be presented to T cells and set off a series of effects (Woo et al., 2015; Altorki et al., 2019). However, in observing individuals, the progression of cancer continues, arguing that the neoplasm finally escaped from the hunting of the immune system. This phenomenon involves the close cooperation of cells and molecules in the immune system.

The initial recognition of neoantigens starts with antigen exposure and uptake, which is accomplished by dendritic cells (DCs). The immigrated DCs present antigen peptides and promote the activation of adaptive immune cells, such as CD8^+^ T cells. The cascade activated CD8^+^ T cells flow back to the tumor microenvironment (TME), recognizing and attacking malignant cells. Simultaneously, other innate immune cells, also participate in the TME, including natural killer cells (NK cells), γδT cells, plasmacytoid DCs, macrophages, monocytes, and some myeloid cells (Ribas, 2015; Patel and Minn, 2018). Different kinds of innate immune cells comprise a great network to respond to the emergence of neoantigens.

Some macrophages are described as essential populations of cells in immune-oncology. The conventional recognized macrophages that are involved in the inflammatory response and act as antimicrobic and antitumor components of innate immunity are classified as M1 macrophages. M2 macrophages often have alternative activation, and function as immune modulators in Th2-related mechanisms and cancer development (Allavena et al., 2008; Mantovani and Locati, 2016). The term “tumor-associated macrophages” (TAMs) refers to macrophages existing in TME and share similar surface markers with M2 macrophages. Scientists have proposed that TAMs do not represent a subset of macrophages owing to their instability and dependence on tumors.

Myeloid-derived suppressor cells (MDSCs) comprise a group of myeloid cells generated in pathology, mainly cancer. The most remarkable proposed function of MDCSs is as a suppressor of T cell response. MDSCs are often closely involved in the progression of metastasis in different kinds of malignancies. Tumor cells secrete growth factors and chemokines to expand and recruit MDSCs, which play a role in protecting tumor cells from destruction by host immunity and physical injury, facilitate immune escape and support angiogenesis in neoplasms (Gabrilovich and Nagaraj, 2009; Talmadge and Gabrilovich, 2013; Veglia et al., 2018).

## Latest Achievements in lncRNAs Modulation in Immune-Oncology

**FIGURE 3:**
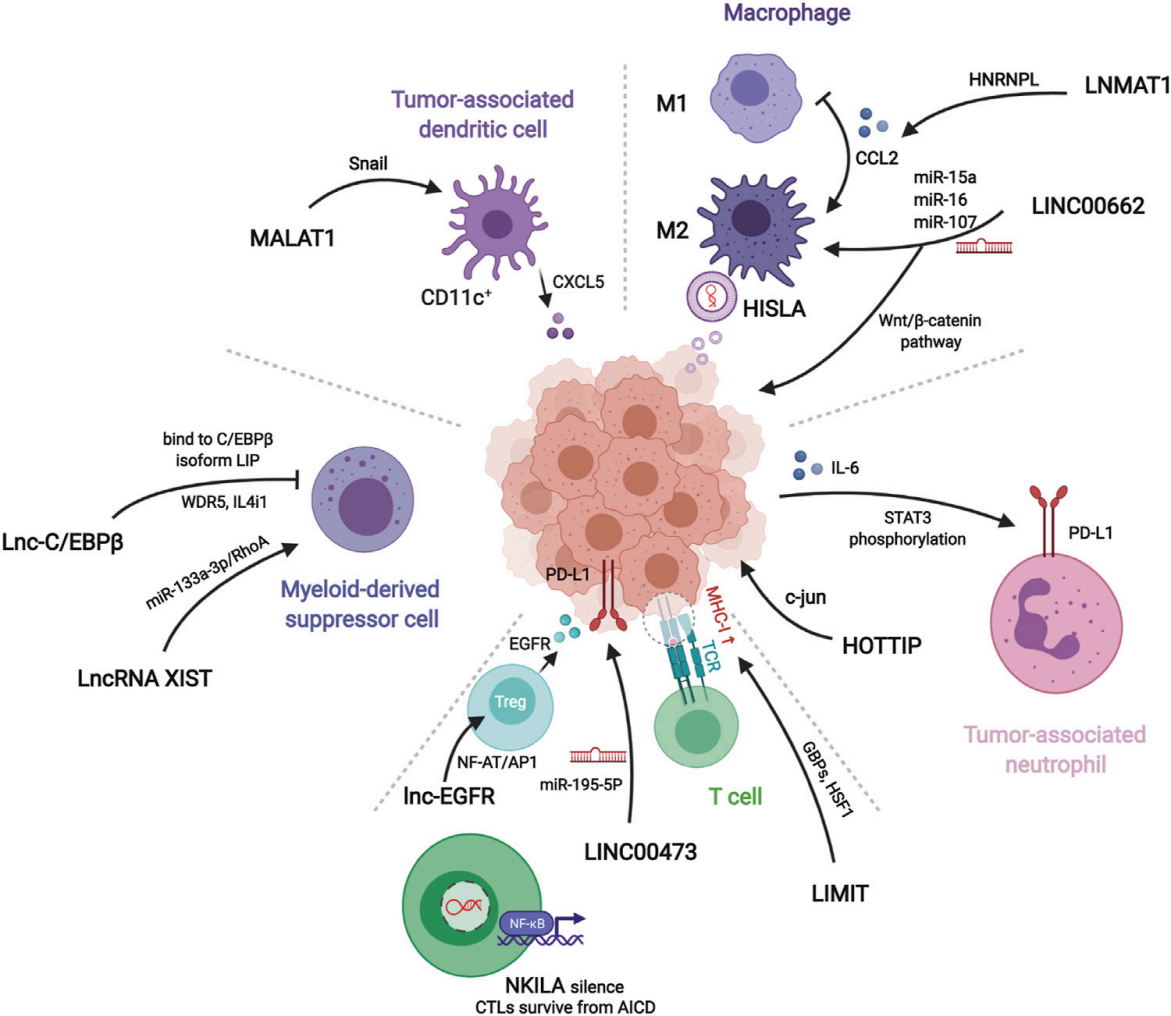
Mechanisms of lncRNAs related to different immune components in tumor microenvironment. LNMAT1, LINC00662, and HISLA regulate macrophages polariztion or tumor growth through chemokine, wnt pathway, or extracellular vesicles, respectively. HOTTIP stimulates tumor cells to secret IL-6 for inducing PD-L1 on the surface of tumor-associated neutrophils. LIMIT, LINC00473, NKILA, and lnc-EGFR modulate recognition and cytotoxicity of T cells to tumor cells. Myeloid-derived suppressor cells and tumor-associated dendritic cells assist tumor immune evasion by lnc-C/EBPβ, lncRNA XIST, and MALAT1.

Here, we retrieved all relative original studies in PubMed (www.ncbi.nlm.nih.gov/pubmed). The Medical Subject Headings (MeSH) terms used were “RNA, Long Noncoding” AND “Neoplasms” AND cell types, containing “Macrophages” (51 results), “Dendritic Cells” (5 results), “Killer Cells, Natural” (4 results),” Neutrophils” (6 results), “Myeloid-Derived Suppressor Cells” (8 results), “T-Lymphocytes” (49 results), “B-Lymphocytes” (20 results) and “Lymphocytes, Tumor-Infiltrating” (14 results). Ultimately, all results were checked artificially. We only enrolled the lncRNA research referring to the immune effects confirmed by cell and/or animal oncological experiments. Studies on lymphomas caused by immune cell development disorders were eliminated (Table 1; Figure 3).

**TABLE 1:** Research on lncRNAs as regulators in cancer immunity.

LncRNA	Immune component	Mechanism	Function	Reference
HISLA	Macrophage	Stabilize HIF-1α in cancer cells via extracellular vesicle	Upregulate aerobic glycolysis in breast cancer cells	Chen et al. (2019)
CamK-a	Macrophage	Activate NF-κB pathway through Ca^2+^ signaling	Remodel tumor microenvironment and recruit macrophages	Sang et al. (2018)
Lnc-BM	Macrophage	Bind and regulate JAK2/STAT3 pathway, express CCL2	Recruit macrophages and promote brain metastasis in breast cancer	Wang et al. (2017)
LNMAT1	Macrophage	Recruit hnRNPL to promoter and upregulate expression of CCL2	Recruit macrophages and promote lymphatic metastasis of bladder cancer	Chen et al. (2018)
LncRNA-MM2P	Macrophage	Reducing phosphorylation of STAT6 and regulate secretion of cytokines	Promote M2 macrophages polarization	Cao et al. (2019)
H19	Macrophage	Upregulate activation of miR-193b/MAPK axis induced by macrophages	Promote cell aggressiveness in hepatocellular carcinoma	Ye et al. (2020)
LINC00662	Macrophage	Activate Wnt/β-catenin signaling	Promote M2 macrophages polarization and hepatocellular carcinoma progression	Tian et al. (2020a)
RPPH1	Macrophage	Interact with TUBB3 mediated by exosomes	Promote M2 macrophages polarization and colon cancer metastasis	Liang et al. (2019)
MALAT1	Macrophage	Activate STAT3/MALAT1 pathway mediated by M2 macrophages secreted IL-8	Promote tumorigenesis of prostate cancer	Zheng et al. (2018)
JHDM1D-AS1	Macrophage	Increase the formation of CD31^+^ blood vessels	Promote infiltration of CD11b+ macrophages and tumor growth	Kondo et al. (2017)
lncRNA cox-2	Macrophage	Decrease the expression of 1L-10, iNOS, and TNF-α in M1 macrophages	Reduce cell proliferation invasion, EMT, and angiogenesis in hepatocellular carcinoma	Ye et al. (2018)
MALAT1	Macrophage	Modulate FGF2 protein secreted by tumor-associated macrophages	Promote angiogenesis of thyroid cancer	Huang et al. (2017)
NIFK-AS1	Macrophage	Act as ceRNA of miR-146a	Inhibit M2 polarization of macrophages	Zhou et al. (2018b)
Xist	Macrophage	Suppress the expression of IL-4, mediated by TCG-4	Promote M2 polarization of macrophages and progression of lung cancer	Sun and Xu, (2019)
ANCR	Macrophage	Regulate expression of FoxO1	Inhibit M1 polarization of macrophages and promote invasion and migration of gastric cancer	Xie et al. (2020)
CCAT1	Macrophage	Act as ceRNA via CCAT1/miR-148a/PKCζ regulation	Inhibit M2 polarization of macrophages and migration of prostate cancer	Liu et al. (2019)
UCA1	Macrophage	Upregulate protein levels of p-AKT	Promote invasiveness of breast cancer cell	Chen et al. (2015)
LOC100129620	Macrophage	Promote IL-10 expression in osteosarcoma cells	Promote M2 polarization of macrophages, proliferation, angiogenesis of osteosarcoma	Chen et al. (2021b)
LINC01140	Macrophage	Act as ceRNA via LINC01140/miR-140-5p/FGF9 axis	Promote aggressiveness and macrophage M2 polarization of bladder cancer cell	Wu et al. (2020a)
LINC00514	Macrophage	Upregulate Jagged1-mediated notch signaling pathway	Promote M2 polarization and metastasis of breast cancer	Tao et al. (2020)
PCAT6	Macrophage	M2 macrophages secret VEGF to stimulate the upregulation of PCAT6 in breast cancer cell	Promoting angiogenesis in triple-negative breast cancer	Dong et al. (2020)
LincRNA-p21	Macrophage	MDM2 eliciting proteasome-dependent regulation to p53/NF-κB/STAT3 pathway	Promote M2 polarization and progression of breast cancer	Zhou et al. (2020)
RP11-361F15.2	Macrophage	Act as ceRNA via RP11-361F15.2/miR-30c-5p/CPEB4 axis	Promote M2 polarization and tumorigenesis of osteosarcoma	Yang et al. (2020)
SNHG15	Macrophage	Act as SNHG15/CDK6/miR-627 circuit by palbociclib	Promote M2 polarization of glioma associated microglia in glioblastoma multiforme	Li et al. (2019)
HOTTIP	Neutrophil	Enhance IL-6 expression	Upregulate the expression of PD-L1 in neutrophils to potentiate immune escape of ovarian cancer cells	Shang et al. (2019)
LINC01116	Neutrophil	Enhance DDX5-mediated IL-1β expression in glioma cell	Promote tumor proliferation and tumor-associated neutrophils recruitment	Wang et al. (2020a)
MALAT1	Dendritic cell	Upregulate expression of Snail and activate functions of CCL5	Promote colon cancer progression	Kan et al. (2015)
Pvt1	MDSC	Regulate the downstream functions of G-MDSC	Enhance suppressive immunity in tumor microenvironment	Zheng et al. (2019)
Olfr29-ps1	MDSC			
Lnc-C/EBPβ	MDSC	Regulate the activity of transcripts, such as COX2, NOX2, NOS2, and Arg-1	Suppressive functions of MDSCs	Gao et al. (2018a)
Xist	MDSC	Act as ceRNA via miR-133a-3p/RhoA regulation	Promote inflammation-driven colorectal cancer progression	Yu et al. (2019)
Lnc-chop	MDSC	Interact with CHOP and the C/EBPβ isoform liver-enriched inhibitory protein	Regulate impressive functions MDSCs in TME	Gao et al. (2018b)
Lnc-C/EBPβ	MDSC	Regulate IL4i1 mediated by C/EBPβ LIP and WDR5	Modulate differentiation of MDSCs	Gao et al. (2019)
MALAT1	MDSC	Upregulate of Arg-1 and increase proportions of MDSCs	Inhibit Immunosuppression in lung cancer	Zhou et al. (2018a)
RNUXOR	MDSC	Bind with RUNX1 and increase levels of Arg-1 in MDSC	Immunosuppression in lung cancer	Tian et al. (2018)
AK036396	MDSC	Enhance stability of Ficolin B	Inhibit maturation and accelerate immunosuppression of PMN-MDSCs in lung cancer	Tian et al. (2020b)
NKILA	T cell	Modulate activated-induced cell death via NKILA activity mediated by Ca2+	Sensitizing T cells and promote tumor immune evasion	Huang et al. (2018)
lnc-EGFR	T cell	Protect EGFR from ubiquitination and activate AP-1/NF-AT1 pathway	Stimulate differentiation of Tregs and promote immune evasion in hepatocellular carcinoma	Jiang et al. (2017)
Flicr	T cell	Regulate transcription of Foxp3 mediated by modifying chromatin accessibility in CNS3/AR5 region of Foxp3	Enhance immune escape dominated by Tregs	Zemmour et al. (2017)
Lnc-Tim3	T cell	Bind to Tim-3 and induce nuclear translocation of Bat3	Exacerbate CD8^+^ T cell exhaustion	Ji et al. (2018)
SNHG1	T cell	Act as ceRNA via miR-448/IDO regulation	Regulate Tregs differentiation and affect immune escape of breast cancer	Pei et al. (2018)
MALAT1	T cell	Act as ceRNA mediated by miR-195	Promotes tumorigenesis and immune escape of diffuse B cell lymphoma	Wang et al. (2019)
Lnc-sox5	T cell	Upregulate expression of IDO1 and modulate infiltration and cytotoxicity of CD3^+^CD8^+^ T cells	Promote progression in colorectal cancer	Wu et al. (2017)
NEAT1	T cell	Act as ceRNA via miR-155/Tim-3	Enhance the antitumor activity of CD8^+^ T cell against hepatocellular carcinoma	Yan et al. (2019)
LINC00473	T cell	Act as ceRNA mediated by miR-195-5p/PD-L1 regulation	Modulate the activation of CD8^+^ T cells for attacking cancer cells	Zhou et al. (2019)
LIMIT	T cell	Upregulate the LIMIT-GBP-HSP1 axis to boost MHC-I, but not PD-L1	Promote tumor antigen recognition and T cells infiltration	Li et al. (2021)
UCA1	T cell	Upregulate the miR-148a/PD-L1 pathway in tumor cells	Attenuate the killing effect of cytotoxic CD8 + T cells on anaplastic thyroid carcinoma cells	Wang et al. (2021b)
NNT-AS1	T cell	Upregulate the TGF-β signaling pathway	Decrease tumor CD4 lymphocyte infiltration in hepatocellular carcinoma	Wang et al. (2020b)
LINC00301	T cell	Upregulate the TGF-β signaling pathway via the FOXC1/LINC00301/HIF1α pathways	Triggers an immune-suppressing microenvironment in non-small cell lung cancer	Sun et al. (2020a)
LINC00473	T cell	Upregulate PD-L1 via LINC00473/miR-195-5p	Suppress the activation of CD8^+^ T cell	Zhou et al. (2019)
LINC00240	Natural killer T cell	Induction of miR-124-3p/STAT3/MICA-mediated NKT cell tolerance	Suppress natural killer T cell cytotoxic activity in cervical cancer	Zhang et al. (2020a)

### Macrophages

In all articles retrieved, three classes of mechanisms of macrophages engaging in the immune modulation of tumor behavior were identified. Studies on macrophage polarization reported the most numerous RNA molecules. The epigenetic regulation implemented by lncRNAs influences the expression of CCL2, which is prominent in macrophage recruitment. The interaction of glucometabolism between TAM and cancer cells provides an interesting method for signaling communication via extracellular vesicles (EVs).

LINC00662 is identified as a tumor-promoting marker because of its involvement in patient’s prognosis in hepatocellular carcinoma (HCC). Its endogenous competing mechanism explains its behavior in cytoplasm. During competitive binding with miR-15a, miR-16, and miR-107, LINC00662 activates the expression and secretion of WNT3A. By the way of autocrine and paracrine, both in HCC cells and macrophages upregulate the Wnt/β-catenin pathway to promote cancer cell migration and macrophage polarization in TME, respectively (Tian et al., 2020a).

The lncRNA, LNMAT1, is highly expressed in tissue from lymph nodes of bladder cancer patients, which is significantly related to their prognosis and tumor metastasis. LNMAT1, as an intergenic gene, can dramatically enhance the transcription of CCL2, by recruiting hnRNPL to its promoter and mediatingH3K4 tri-methylation. The silenced cells demonstrate that CCL2 is the essential protein inducing LNMAT1-related lymph node metastasis. CCL2 enrolled macrophages can lead to TAM infiltration and lymph angiogenesis (Chen et al., 2018).

Lnc-BM also affects metastasis by CCL2 function of recruiting macrophages. A study of breast cancer brain metastases showed that the interaction between Lnc-BM and JAK activates the latter and results in phosphorylation of STAT3, of which the downstream proteins contain ICAM1 and CCL2. The consecutive action of Lnc-BM/JAK/STAT3 pathway prompts the vascular co-option and ultimately induces macrophages migration and penetration of the blood-brain barrier. Notably, the critical factors IL-6 and endostatin M, which trigger STAT3 phosphorylation, can in return be secreted by recruited macrophages. The mechanism shows a positive feedback loop in cytokines action (Wang et al., 2017).

HISLA is a myeloid-specific molecule, which is packaged in the EV secreted from TAMs of breast cancer cells. The extracellular vesicle has been reported as an efficient transporter in TME, carrying critical signaling molecules (Luga et al., 2012; Boelens et al., 2014). The transmission of HISLA from TAM to cancer cells restrains the hydroxylation via a reduction of the binding between PHD2 and HIF-1α, and stabilizes the latter (Semenza, 2012; Semenza, 2013). The relatively high level of the oxygen-sensing transcription factor, HIF-1α, enhances aerobic glycolysis and the capability of apoptosis resistance in breast cancer cells. Correspondingly, the accumulation of lactate up-regulates the abundance in cancer cells, which creates a positive feedback loop between interactions of TAMs and cancer cells, leading to the survival of cancer cells under stress and therapy. Finally, the resistance of chemotherapy was validated in a mouse model experiment (Chen et al., 2019).

### Neutrophils

Tumor-associated neutrophils are cells in the TME that have gradually attracted attention, which can be recruited to regulate adaptive immunity through the PD-1/PD-L1 pathway (He et al., 2015). HOTTIP, a long noncoding RNA, promotes the secretion of IL-6 in ovarian cancer cells, maintaining high levels of PD-L1 on the surface of neutrophils. Consequently, the accumulating immune exhaustion inhibits the function of T cells and causing tumor cells to escape immune surveillance (Shang et al., 2019).

### Dendritic Cells

MALAT-1 is a well-known lncRNA with a long history in epigenetic research (Ji et al., 2003). The latest studies report that MALAT1 expressed by tumor associated DCs is attributable to the epithelial-to-mesenchymal transition (EMT), invasion, and migration of tumor cells in colon cancer. Upregulation of MALAT-1 stimulates the expression of Snail, and subsequently activates the functions of CCL5. The downstream manner of CCL5 also refers to proliferation, angiogenesis, and chemotherapeutic resistance, as reported in other oncological studies (Kan et al., 2015; Aldinucci and Casagrande, 2018).

### MDSCs

Studies have shown that MDSCs modulate immune responses in the TME. Lnc-C/EBPβ has been demonstrated as a suppressor in functional regulation of MDSCs. The functioning of lnc-C/EBPβ depends on a series of transcripts, including COX2, NOX2, NOS2, and Arg-1. The downstream targets of these molecules cover the secretion of IFN-γ, the distribution of CD4^+^ and CD8^+^ T cells, and the differentiation of MDSCs. Mechanically, the binding of lncRNA with C/EBPβ (located at the LIP isoform) obstructs the activation of the protein (Gao et al., 2018a).

Other researchers describe different effects of Lnc-C/EBPβ on the subset transformation of CD11b^+^Ly6C^hi^Ly6G^−^ monocytic MDSC (Mo-MDSC) and CD11b^+^Ly6C^low/neg^Ly6G^+^ polymorphonuclear MDSC (PMN-MDSC). *In vitro* and *in vivo* experiments demonstrate that Lnc-C/EBPβ induces bias of PMN-promotion and Mo-suppression during development. This tendency occurs by a two-way method: One is the aforementioned bounding to LIP, and the other is interaction with WDR5 to stop the activation of H3K4me3 in the promoter of IL4i1. The distinct functions of the two subsets of MDSC lead to different influences on neighboring tumor cells (Gao et al., 2019).

Transgenic mouse model experiments showed that LncRNA XIST acts as a regulator of the CXCL12/CXCR4 axis. Higher levels of LncRNA XIST bind with miR-133a-3p and functioning as molecule sponge, liberating the block of RhoA by miRNA. These actions promote the EMT, recruitment of MDSCs and macrophages, and accumulation of antigen-presenting cells, contributing to tumorigenesis under a continuous inflammatory environment (Yu et al., 2019).

### T Cells

In cancer immunosurveillance, cytotoxic T lymphocytes are dominant in the identification and destruction of cancer cells. However, for the existence of regulation factors, such as TNF, FasL, and TRAIL, activated T cells in TME often suffer immunological elimination, called activation-induced cell death (AICD) (Schietinger and Greenberg, 2014). NKILA, an lncRNA translated in T cells, sets off the activating threshold of AICD. Under the status of T cell activation, the Ca^2+^ related signal removes the deacetylase in area of *NKILA* promoter and up-regulates the transcription mediated by STAT1. Treatment of patient-derived xenografts (PDXs) of breast cancer with NKILA-CTLs injection has shown compelling efficiency in suppressing tumor growth. This study indicates the stirring potential strategies of immunotherapy of lncRNAs engineering (Huang et al., 2018).

The promotion of differentiation of regulatory T cells (Tregs) is also a subject of T cell studies in cancer immunity. Lnc-EGFR can combine with the target transcript EGFR in a sequence-specific manner and protect it from ubiquitination mediated by c-CBL. Stabilization of EGFR sets up the AP-1/NF-AT1 pathway, which consists of positive feedback enhancing the expression of EGFR. The modulation of lnc-EGFR was confirmed in both PDX and humans with HCC, and results in Treg differentiation, CTL suppression, and tumor growth (Jiang et al., 2017).

The co-stimulation signals between activated T cells and tumor cells have been proposed as a convincing target of immune therapy. LINC00473 has been identified as a regulator of programmed death-ligand-1 (PD-L1) in pancreatic cancer. The RNA molecule works as an ceRNA, buffering the levels of miR-195-5p and mRNA of PD-L1. The results demonstrated that silencing of LINC00473 contributes to enhanced expression of IFN-γ, Bax, and IL-4, and, simultaneously, decreases the levels of MMP-2, MMP-9, IL-10, and Bcl-2. Thus, LINC00473 induces a reduction of apoptosis and enhanced proliferation, invasion, and migration of cancer cells (Zhou et al., 2019).

Major histocompatibility complex-I (MHC-I) represents a type of molecule that presents antigens to CD8^+^ T cells, involving tumor antigen recognition. The lncRNA, inducing MHC-I and immunogenicity of tumor (LIMIT) acts as an accelerator in MHC-I generation. LIMIT can be stimulated by IFN-γ and cis-activate the guanylate-binding protein (GBP) gene cluster. GBPs can disrupt the interaction between HSP90 and HSF1, followed by transcriptional activation of MHC-I, but not PD-L1. This finding indicates a potential pan-cancer epigenetic target of immune therapy (Li et al., 2021).

### Clinical Translational Research

Exploring the mechanisms of lncRNAs provides new insights into applications of the molecules as drugs and biomarkers. As this is still an emerging field, only a handful of lncRNA drugs have been approved, but there is accumulated experience with RNA drugs. Small interfering RNAs (siRNAs), antisense oligonucleotides (ASOs), and the CRISPR/Cas9 system are commonly used in drug design (Matsui and Corey, 2017; Chen et al., 2021a). Malat1, a well-studied lncRNA, was systemically knocked down using ASOs in a mouse mammary carcinoma model, and showed lower tumor burden and significant reduction in metastasis. Given the roles of the drugs acting on cancer immunity have not been well elucidated, and their efficacy and safety determined through lnRNAs primary studies will stimulate confidence for them to be tested in clinical trials (Arun et al., 2016). In the meantime, bioinformatics is used in an attempt to filter out the vital lncRNAs in cancer immunity of different kinds of malignancies (Wang et al., 2018; Sun J. et al., 2020; Wu Y. et al., 2020; Zhang et al., 2020b; Wang J. et al., 2021; Mao et al., 2021; Zhou et al., 2021), and the findings may help with biomarker construction or further mechanism research. In a clinical trial of efficacy biomarkers based on the phase 2 IMvigor210 cohort, the authors reported a novel lncRNA-based immune classification in cancer immunotherapy and recommended immunotherapy for the immune-active class.

## Conclusion and Perspective

Research on the immunological modulation of lncRNAs in affecting cancer manners is an emerging area enriched by interactive innovation in multiple disciplines. As detailed in the review, lncRNAs engage in many critical events in the immunological balance of elimination or escape in tumor locations. For oncologists, much more attention should be paid to the interactions between cells rather than focusing on single cancer cells and doubling the difficulty. The major challenge to study on lncRNAs is that despite their importance, the current technologies are used to reveal their molecular mechanisms are quite technically difficult. Explaining the mechanism of lncRNA regulation requires the excellent ability to perform epigenetic and molecular biologic experiments. New technologies, for example, new sequencing methods that can directly sequence DNA, RNA, and RNA modifications as well as proteomics will shed light on their role in the transcription processes, immune modulation, and cancer progression. All this technological progress may enhance our understanding of lncRNAs in cancer and will give a better view of disease etiology and will help guide future diagnosis and ultimately therapeutic options. Nevertheless, the possibility of enhancing our understanding of immune surveillance and finding new therapeutic targets for cancer offers continuous motion of relative discovery.
